# Toward a nearly defect-free coating via high-energy plasma sparks

**DOI:** 10.1038/s41598-017-02702-3

**Published:** 2017-05-24

**Authors:** Mosab Kaseem, Hae Woong Yang, Young Gun Ko

**Affiliations:** Materials Electrochemistry Laboratory, School of Materials Science and Engineering, Yeungnam University, Gyeongsan, 38541 Republic of Korea

## Abstract

A nearly defect-free metal-oxide-based coating structure was made on Al-Mg-Si alloy by plasma electrolytic oxidation at high current density accompanying high-energy plasma sparks. The present coatings were performed at two different current densities of 50 and 125 mA/cm^2^ in the alkaline-phosphate-based electrolytes with different concentrations of sodium hexafluoroaluminate (Na_3_AlF_6_). The addition of (Na_3_AlF_6_) to the electrolyte used in this study would result in a decrease in the size of the micropore, and a reasonably defect-free coating structure was achieved in the sample treated at high current density of 125 mA/cm^2^. This was attributed mainly to the hydrolysis of AlF_6_^3−^ triggered by intense plasma sparks, which resulted in a uniform distribution of fluorine throughout the coating. Accordingly, the corrosion performance of the coating formed in the electrolyte containing 1.5 g/L Na_3_AlF_6_ at 125 mA/cm^2^ was improved significantly as confirmed by electrochemical impedance analysis. In addition, the formation mechanism of the nearly defect-free coating in the presence of Na_3_AlF_6_ was discussed.

## Introduction

In recent years, a plasma electrolytic oxidation (PEO) has emerged as a novel method for surface treatment capable of producing metal-oxide-based coatings with desirable properties on the surface of valve metals and alloys^1–6^. When a high voltage above the breakdown voltage would be applied, a protective coating formed on the surface of the metal substrate by plasma-assisted electrochemical reactions^7, 8^. Typically, the structure of PEO coating comprised two distinct layers: an outer layer with a number of the micropores, cracks, and structural defects, and an inner layer with a relatively compact structure. The micropores and other defects were distributed inhomogeneously in both layers. They were more evident in the outer layer. This would cause the infiltration of corrosive medium into the inner layer of the coating and, thereby, arrive at the substrate, which accelerated the corrosion rate due to a local change in pH^9^. To alleviate the present problem, considerable attention has been paid to improving the fabrication of defect-free coatings by PEO through the development of novel electrolytic systems together with the optimization of the electrical parameters, such as frequency and current density^8^.

Among various approaches, the incorporations of inorganic particles, such as ZrO_2_, TiO_2_, CeO_2_, and clay, was considered one of the useful strategies, Kaseem *et al*.^10^ suggested an effective way to block the micropores by incorporating ZrO_2_ and MoO_2_ particles into the coating, which improved significantly the corrosion performance of 7075 Al alloy. According to recent results by Rapheal *et al*.^11^, however, the micropores and cracks were still clearly visible in the coating, which might provide the short paths for the corrosive medium although the addition of clay helped increase the compactness of the coating formed on an AM50 Mg alloy by PEO at a low current density of 30 mA/cm^2^. In contrast, Lu *et al*.^12^ demonstrated that, in coating formed by PEO on AM50 Mg alloy, the micropores would be filled fairly by adding Si_3_N_4_ particles to the KOH-Na_3_PO_4_-containing electrolyte. The distribution of the microdefects found in cross section of the coating was still in argument. Thus, the improvement in corrosion performance was case-sensitive.

On the other hand, the incorporation of F^−^ ions into the coatings has been reported as an effective strategy to improve the corrosion performance^13–18^. Per potentiodynamic polarization results reported by Kazanski *et al*.^13^, the coating formed on AZ91 Mg alloy by PEO under alternating current in an alkaline silicate-based electrolyte containing KF exhibited better corrosion performance as compared to the counterpart coating formed without KF. This result suggested that a significant drop in the porosity was achieved by the incorporation of F^−^ ions into the coating. Indeed, Duan *et al*.^18^ suggested that KF was able to enhance the corrosion resistance of the inner layer. It was, therefore, concluded that F^−^ ions would improve the corrosion performance due to the formations of F-compounds that would increase the thickness and compactness of the inner layer. In case of Mg alloy, MgF_2_ would precipitate readily on the surface of the anode, preventing excessive dissolution of Mg element from the substrate^8, 14^. However, Yerokhin *et al*.^19^ reported that the addition of F^−^ ions to the electrolyte showed a different tendency of Al-oxide coating. Sodium hexafluoroaluminate (Na_3_AlF_6_) was prone to be hydrolyzed in aqueous solution, resulting in the formations of aluminum hydroxide (Al(OH)_3_) and F-containing compounds. Hence, the observations in the previous studies raised the question of whether the use of the complex fluorine-containing salts would be desirable for fabricating a nearly defect-free coating via PEO because the hydrolysis of Na_3_AlF_6_ might lead to the structural modification of the coating. Up to the present study, the fabrication of a nearly defect-free coating on Al-based alloy under high-energy plasma condition using the fluorine-containing salt as an additive in the electrolyte and the electrochemical contribution of the present coating have rarely been understood.

Therefore, the aim of this work is to fabricate the nearly defect-free compact coating on Al-Mg-Si alloy by PEO to improve corrosion performance by adding Na_3_AlF_6_ to the alkaline phosphate-based electrolyte. On the basis of the results, the mechanism on the formation of the coatings in the presence of Na_3_AlF_6_ will be discussed in relation to the structural characteristics controlled with respect to current density as well as Na_3_AlF_6_ concentration.

## Results and Discussion

### Structural characteristics of coating

**Figure 1:**
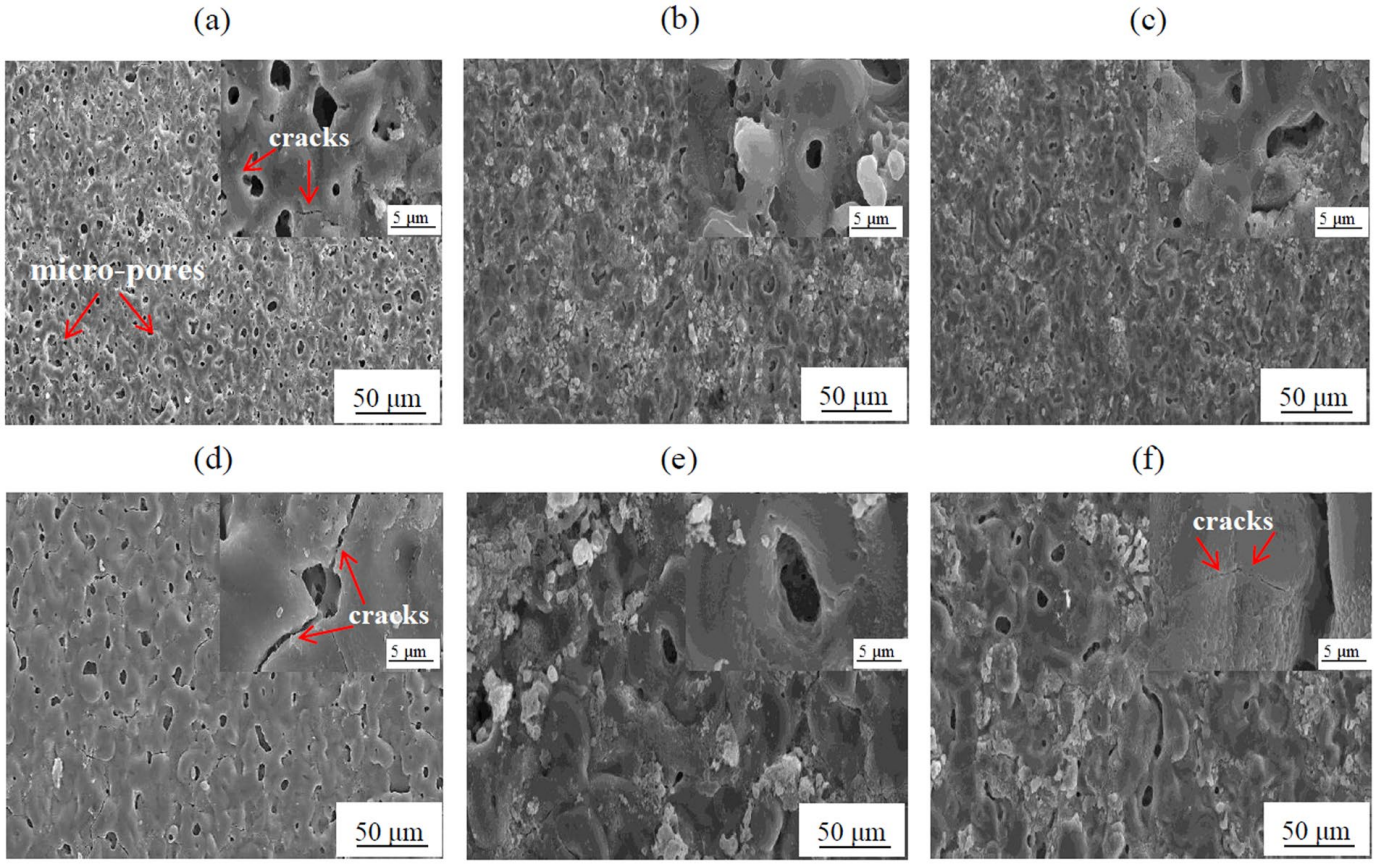
SEM images showing the surface morphology of the coatings formed on an Al-Mg-Si alloy by PEO at 50 mA/cm^2^ (**a**–**c**) and 125 mA/cm^2^ (**d**–**f**) in an electrolyte containing no Na_3_AlF_6_ (**a** and **d**), 1.5 g/L Na_3_AlF_6_ (**b** and **e**), and 3 g/L Na_3_AlF_6_ (**c** and **f**). Insets are the high-magnification images of the surface morphology of the PEO coatings.

Figure 1 shows the changes in surface morphology of the PEO coatings as a function of Na_3_AlF_6_ concentration and current density. In all coatings, the surface was dominated by numerous micropores, which are characteristic of PEO coatings. To elucidate the effect of Na_3_AlF_6_ concentration and current density on the surface morphology of the coatings formed by PEO, the porosity and pore size values were measured, and they are listed in Table 1. Irrespective of current density, it was observed that the average size of the micropores and cracks, as well as the degree of porosity, was lower for the coatings formed with 1.5 g/L Na_3_AlF_6_. This suggested that the addition of 1.5 g/L Na_3_AlF_6_ to the phosphate-based electrolyte might decrease the intensity of plasma discharges, resulting in the generation of a compact microstructure with less structural defects (Fig. 1b and e). Indeed, when adding 3 g/L Na_3_AlF_6_ to the electrolyte, the porosity and micropore size increased as compared to the sample obtained in an electrolyte containing 1.5 g/L Na_3_AlF_6_. In addition, from the high-magnification SEM images shown in the insets of Fig. 1, two important observations were made regarding surface morphology. Firstly, as current density increased, the micropore size tended to increase, whereas the percentage of porosity tended to decrease. Secondly, the coating obtained with no Na_3_AlF_6_ at 125 mA/cm^2^ exhibited severe cracking as compared to the coatings formed in electrolytes containing Na_3_AlF_6_, which is consistent with earlier reports^11, 20, 21^. For the coatings formed in the electrolyte with no Na_3_AlF_6_, larger micropores and cracks were formed at the higher current density due to the increased plasma discharge intensity after quenching by the surrounding electrolyte. However, interestingly, for the coatings formed in electrolyte containing Na_3_AlF_6_, the number of cracks tended to decrease as current density increased, suggesting that the addition of Na_3_AlF_6_ together with a higher current density could promote the incorporation of F-containing compounds, thus decreasing the size of micropores and cracks (Fig. 1e and f).

**Table 1:** Pore size and porosity of the PEO coatings formed on an Al-Mg-Si alloy by PEO for 8 min using different concentrations of Na_3_AlF_6_ at different current densities.

Sample	50 mA/cm^2^			125 mA/cm^2^		
	0	1.5	3	0	1.5	3
Pore size (μm)	4.30	3.65	4.10	4.89	4.63	5.92
Porosity (%)	8.31	6.20	6.85	4.55	1.19	2.41

Table 2 lists the EDS results corresponding to the surface of the coatings obtained in different electrolytes at different current densities. Irrespective of current density, EDS results showed that the coatings formed in the electrolyte with no Na_3_AlF_6_ were mainly composed of Al, O, and P, while as expected, F was only detected in the coatings formed in electrolytes containing Na_3_AlF_6_. This result indicated that AlF_6_^3−^ ions effectively contributed to form the coating layers during the plasma-assisted electrochemical reaction. It is worth noting that the amount of P was observed to decrease in the coatings formed in electrolytes containing Na_3_AlF_6_, which was attributed to the fact that AlF_6_^3−^ ions prevented the adsorption of phosphate groups on the surface^22^.

**Table 2:** EDS results of the PEO coatings formed on an Al-Mg-Si alloy by PEO for 8 min using different concentrations of Na_3_AlF_6_ at different current densities.

Sample	50 mA/cm^2^			125 mA/cm^2^		
	0	1.5	3	0	1.5	3
Al (wt.%)	57.20	35.11	37.77	40.81	28.44	33.40
O (wt. %)	39.73	60.50	56.08	51.84	64.33	57.1
P (wt.%)	3.07	1.78	1.59	7.35	1.22	0.47
F (wt.%)	—	2.61	4.56	—	6.01	9.03

**Figure 2:**
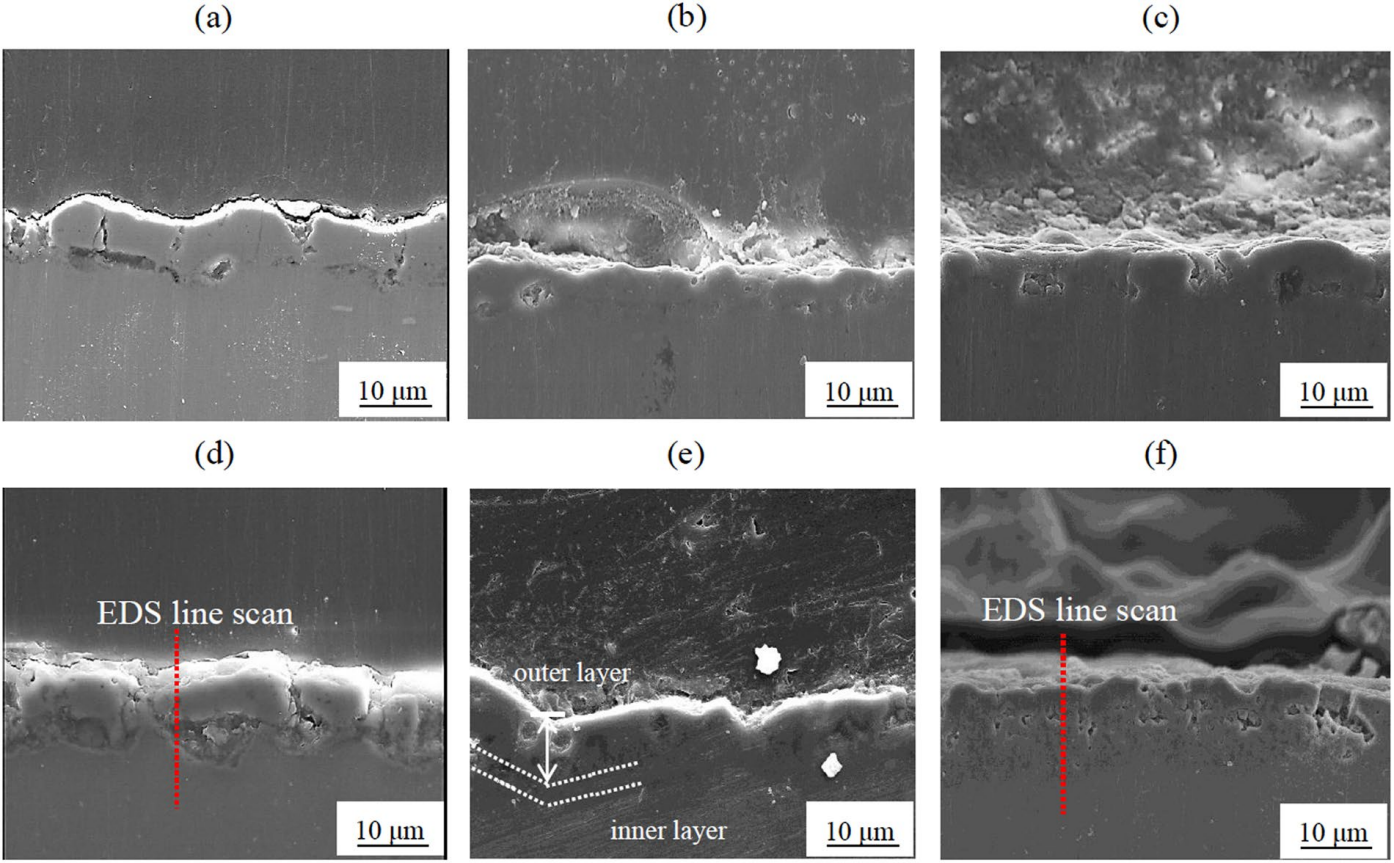
Cross-sectional images of the coatings formed on an Al-Mg-Si alloy by PEO at 50 mA/cm^2^ (**a**–**c**) and 125 mA/cm^2^ (**d**–**f**) in an electrolyte containing no Na_3_AlF_6_ (**a** and **d**), 1.5 g/L Na_3_AlF_6_ (**b** and **e**), and 3 g/L Na_3_AlF_6_ (**c** and **f**).

The cross-sectional images of the PEO coatings obtained in different electrolytes and at different current densities are shown in Fig. 2. It was observed that Na_3_AlF_6_ had no effect on the thickness of the coating. By contrast, the coatings obtained at a current density of 50 mA/cm^2^ exhibited a thickness of about 10 µm, while the coatings obtained at 125 mA/cm^2^ were thicker (with a thickness of ~15 µm). These results were reasonably consistent with those obtained for Ti alloy samples coated by PEO in electrolytes containing different concentrations of calcium hypophosphite^23^. In addition, it was clear that the coatings obtained with Na_3_AlF_6_ exhibited a more compact structure as compared to their counterparts obtained in the electrolyte with no Na_3_AlF_6_, indicating that AlF_6_^3−^ ions decreased the porosity and enhanced the density of the PEO coatings. Since the inner layer of the coatings formed in electrolyte containing Na_3_AlF_6_ was thicker and more compact, it was expected that such coatings would exhibit a higher resistance against corrosion.

**Figure 3:**
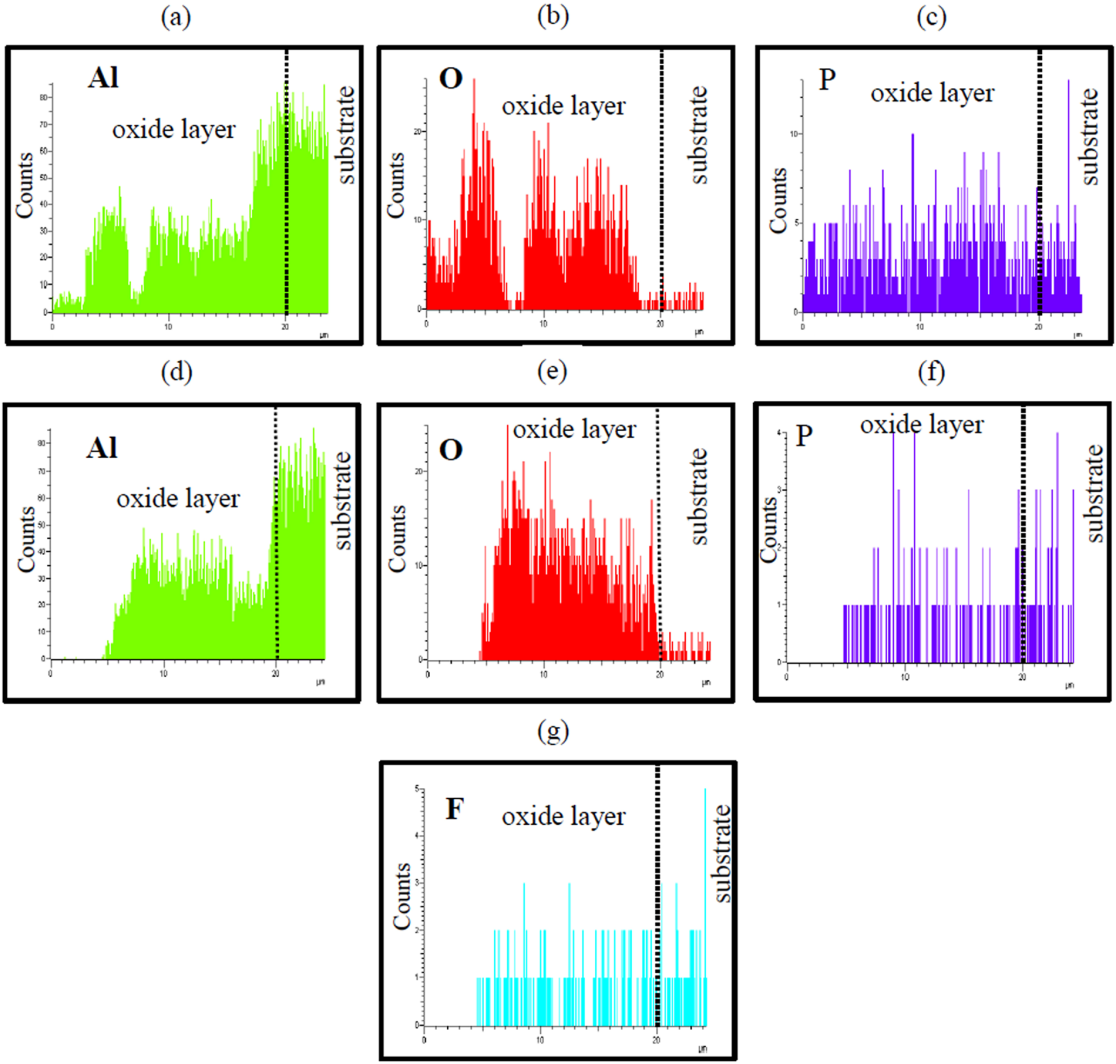
EDS line scan of the cross-section of the coatings formed on an Al-Mg-Si alloy at 125 mA/cm^2^ in electrolyte containing no Na_3_AlF_6_ (**a**–**c**) and 3 g/L Na_3_AlF_6_ (**d**–**g**).

The elemental distribution along the cross-section of the coatings formed at 125 mA/cm^2^ in electrolytes either with no Na_3_AlF_6_ or containing 3 g/L Na_3_AlF_6_ was investigated by EDS line scan (Fig. 3). The EDS analysis of the PEO coating obtained with 3 g/L Na_3_AlF_6_ clearly showed that F was almost uniformly distributed along the cross-section of the coating, while the distribution of P was less uniform as compared to the counterpart coating obtained without Na_3_AlF_6_.

**Figure 4:**
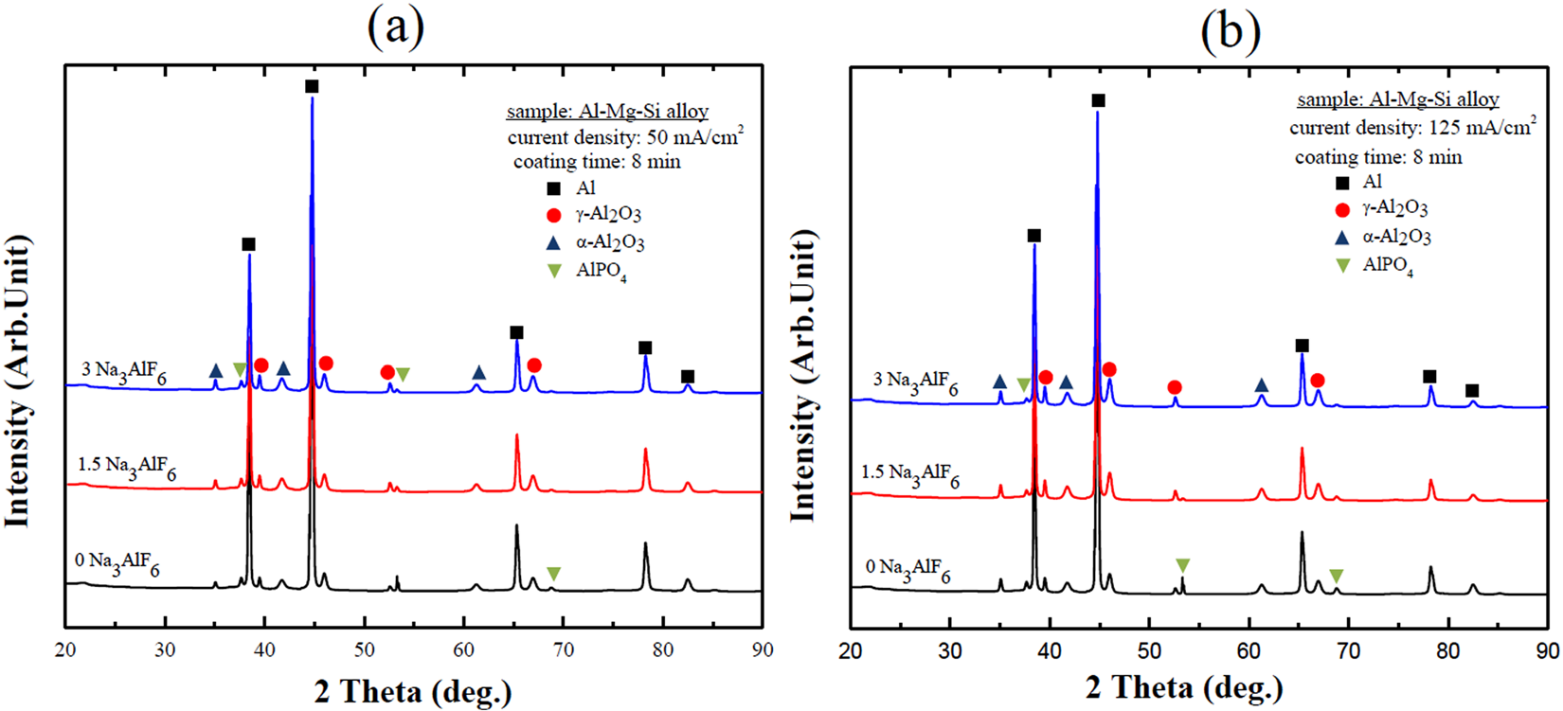
XRD patterns of the coatings formed on al Al-Mg-Si alloy by PEO at (**a**) 50 mA/cm^2^ and (**b**) 125 mA/cm^2^ using electrolytes containing different concentrations of Na_3_AlF_6_.

Figure 4 shows the XRD patterns of the PEO coatings obtained in different electrolytes at different current densities. The XRD peaks corresponding to Al, γ-Al_2_O_3_, α-Al_2_O_3_, and AlPO_4_ were detected in all coatings. The identification of metallic Al in the pattern of the PEO coatings was ascribed to porosity, which caused X-ray penetration through the Al-Mg-Si alloy substrate. During PEO, Al^3+^ ions were released from the substrate and reacted with O^2−^ ions in the electrolyte to produce γ-Al_2_O_3_ and α-Al_2_O_3_ on the surface of the Al-Mg-Si alloy at rapid and slow cooling rates, respectively^24, 25^. On the other hand, AlPO_4_ could be formed by the reaction between PO_4_^3−^ ions, generated through ionization of Na_3_PO_4_ in the electrolyte, and Al^+3^ ions (Equation 1). It is worth noting that although the EDS analysis showed a significant amount of F in the coatings formed in electrolytes containing Na_3_AlF_6_, no peaks attributed to F-containing phases were detected in the XRD patterns, suggesting that F-containing compounds were incorporated into the coatings as the amorphous phase, rather than the crystalline. In addition, it was observed that in the XRD pattern of the coatings formed in electrolyte containing Na_3_AlF_6_, the intensity of all characteristic peaks of γ-Al_2_O_3_ was higher. This suggested that the addition of Na_3_AlF_6_ to the phosphate-based electrolyte affected the phase composition of the coatings. Furthermore, for all coatings, the amount of α-Al_2_O_3_ tended to be lower as compared to that of γ-Al_2_O_3_, which was attributed to the fact that the rapid solidification of molten Al_2_O_3_ did not allow the complete transformation from metastable γ-Al_2_O_3_ into stable α-Al_2_O_3_. Therefore, the amount of the α-Al_2_O_3_ phase obtained was lower under these experimental conditions^26^. Finally, according to the XRD and EDS results, the possible reactions during PEO in the different electrolytes are the following:

1 $$A{l}^{+3}+P{{O}_{4}}^{-3}\to AlP{O}_{4}$$

2 $$Al{{F}_{6}}^{3-}+3{H}_{2}O\to Al{(OH)}_{3}+3{H}^{+}+6{F}^{-}$$

3 $$Al{(OH)}_{3}\,\mathop{\to }\limits^{ \sim 723K}\,\gamma -A{l}_{2}{O}_{3}$$

4 $$Al{(OH)}_{3}\,\mathop{\to }\limits^{ \sim 1373K}\,a-A{l}_{2}{O}_{3}$$

### Electrochemical behavior of coating

**Figure 5:**
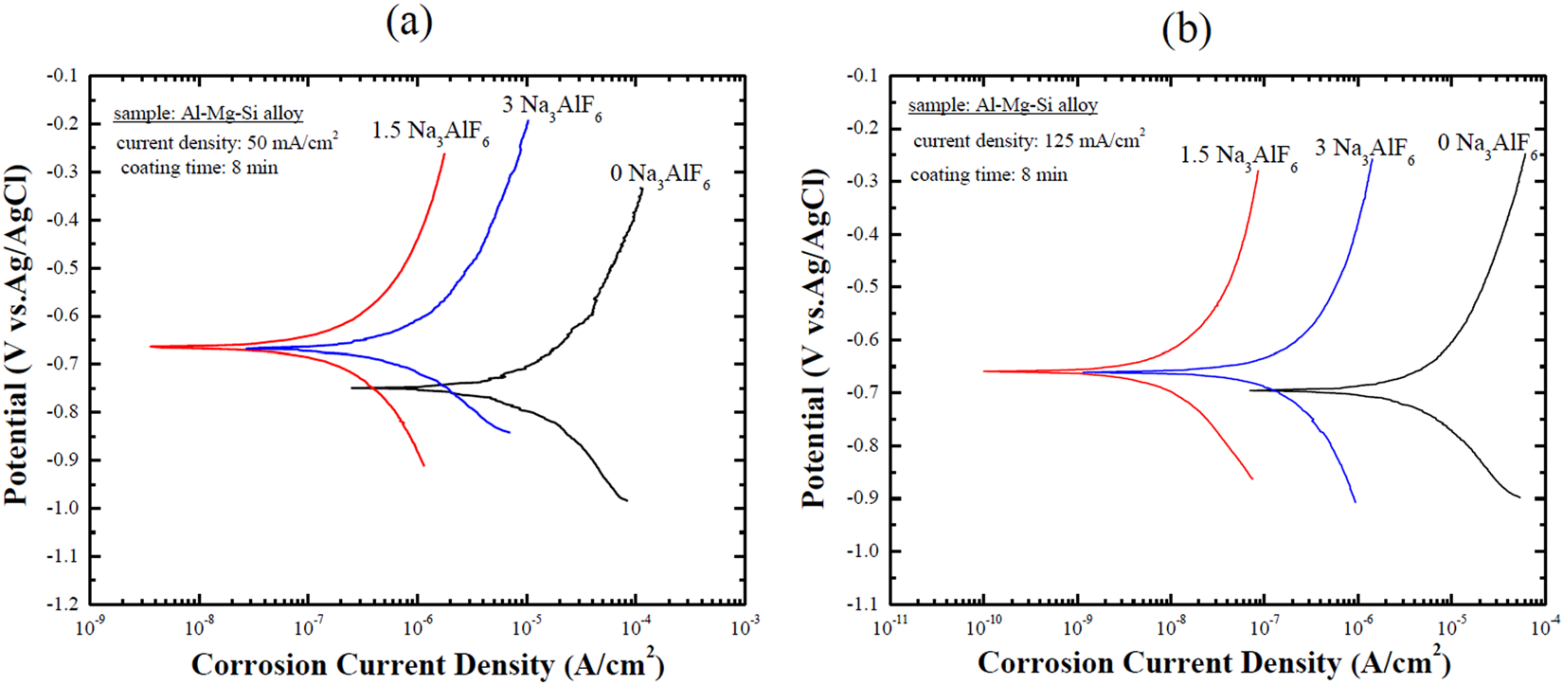
Potentiodynamic polarization curves recorded in 3.5 wt.% NaCl solution of the coatings formed on an Al-Mg-Si alloy by PEO at (**a**) 50 mA/cm^2^ and (**b**) 125 mA/cm^2^ using electrolytes containing different concentrations of Na_3_AlF_6_. The potentiodynamic polarization curves were recorded at a scan rate of 1 mV/s from −0.25 V to +0.4 V with respect to the open circuit potential.

The corrosion resistance of the PEO coatings formed in various electrolytes at two different current densities was evaluated by potentiodynamic polarization tests, and curves are shown in Fig. 5. Corrosion parameters such as the corrosion potential (*E*_*corr*_) and corrosion current density (*i*_*corr*_) were extrapolated from the polarization curves, and the results are listed in Table 3. These results indicated that all coatings obtained in electrolytes containing Na_3_AlF_6_ exhibited a higher resistance against corrosion as compared to the coatings formed in the electrolyte with no Na_3_AlF_6_. In particular, the coating formed in the electrolyte containing 1.5 g/L Na_3_AlF_6_ at 125 mA/cm^2^ exhibited the lowest *i*_*corr*_ of all. This suggested that at a current density of 125 mA/cm^2^, by adding 1.5 g/L Na_3_AlF_6_ to the electrolyte, the anticorrosion properties of the Al-Mg-Si alloy could be significantly improved with respect to the counterparts obtained in electrolytes containing either no Na_3_AlF_6_ or 3 g/L Na_3_AlF_6_. This improvement of the anticorrosion properties was attributed to the formation of a relatively defect-free structure rich in F, which prevented the diffusion of corrosive ions (Cl^−^) towards the substrate. In other studies, Wang *et al*.^27^ reported based on the coating layers formed on LY12 Al alloy by PEO that the *i*_*corr*_ of the coating decreased from 1.85 × 10^−7^ A/cm^2^ to 8.8 × 10^−8^ A/cm^2^ when the electrolyte consisting of 2 g/L NaF and 8 g/L NaAlO_2_ was used at 80 mA/cm^2^ for 60 min as compared to the counterpart obtained from electrolyte without NaF. On the other hand, Arunnellaiappan *et al*.^28^ found that addition of 4 g/L CeO_2_ into the alkaline-silicate-based electrolyte would decrease the *i*_*corr*_ from 4.2 × 10^−7^ A/cm^2^ to 1.25 × 10^−10^ A/cm^2^ when the PEO coatings were formed on 7075 Al alloy at 150 mA/cm^2^ for 10 min. The present results shown in Table 3 clearly implied that the addition of 1.5 g/L Na_3_ AlF_6_ into the electrolyte together with a high current density condition would beneficial for fabricating compact coatings with excellent corrosion protection properties.

**Table 3:** Extrapolation results of the potentiodynamic polarization curves of the PEO coatings formed on an Al-Mg-Si alloy by PEO for 8 min using different concentrations of Na_3_AlF_6_ at different current densities.

Sample	50 mA/cm^2^			125 mA/cm^2^		
	0	1.5	3	0	1.5	3
*E*_*corr*_ (V)	−0.748	−0.667	−0.665	−0.695	−0.659	−0.662
*i*_*corr*_ (A/cm^2^)	1.57 × 10^−5^	1.81 × 10^−6^	6.52 × 10^−7^	3.99 × 10^−6^	9.40 × 10^−9^	1.69 × 10^−7^

**Figure 6:**
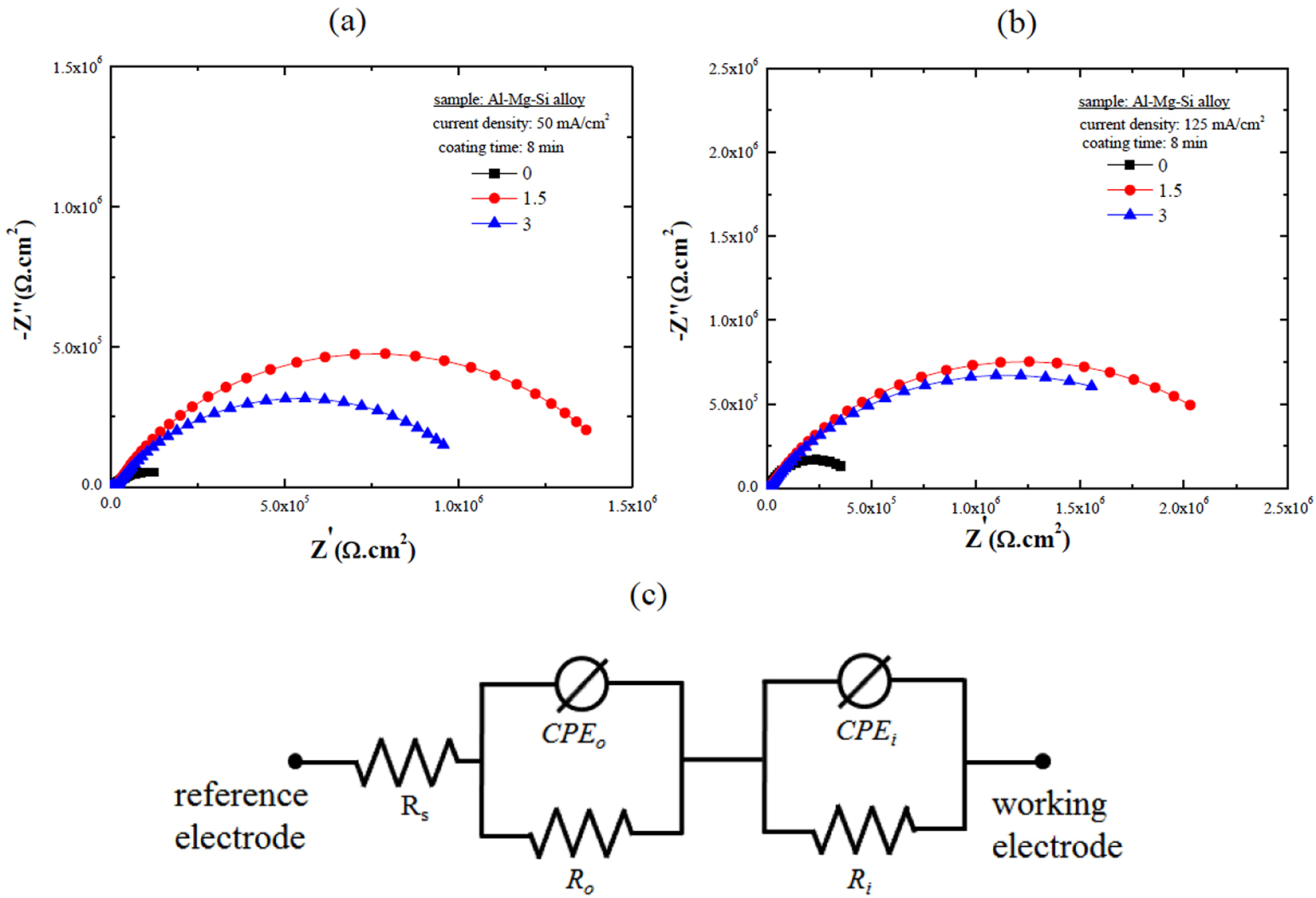
Nyquist plots recorded in 3.5 wt.% NaCl solution of the coatings formed on an Al-Mg-Si alloy by PEO at (**a**) 50 mA/cm^2^ and (**b**) 125 mA/cm^2^ using electrolytes containing different concentrations of Na_3_AlF_6_ and (**c**) the equivalent circuit model used for analyzing the EIS data of the coatings formed in the electrolyte containing Na_3_AlF_6_ with different concentrations at two different current densities, where *R*_*s*_ is the resistance of the solution, *R*_*o*_ and *R*_*i*_ is the resistance of the outer and inner layers of the coatings, respectively, and *CPE*_*o*_ and *CPE*_*i*_ is the constant phase element of the outer and inner layers, respectively.

To investigate the effect of Na_3_AlF_6_ on the corrosion behavior of the Al-Mg-Si alloy coated by PEO in more detail, EIS tests in 3.5 wt.% NaCl solution were conducted, and the results are shown in Fig. 6 as the Nyquist plots. The corrosion resistance of the samples can be qualitatively compared from the EIS spectra, where larger semicircles usually indicate a higher corrosion resistance. The smallest semicircles were observed in the EIS spectrum of the coating formed with no Na_3_AlF_6_, suggesting that the corrosion resistance of the Al-Mg-Si alloy in 3.5 wt.% NaCl solution was improved by the coatings formed in electrolyte containing Na_3_AlF_6_. In addition, irrespective of the current density at which the coating was formed, the coating formed in the electrolyte containing 1.5 g/L Na_3_AlF_6_ exhibited the largest capacitive loop of all, indicating the excellent anticorrosion properties of this coating, which was in good agreement with the potentiodynamic polarization results shown in Fig. 5.

**Figure 7:**
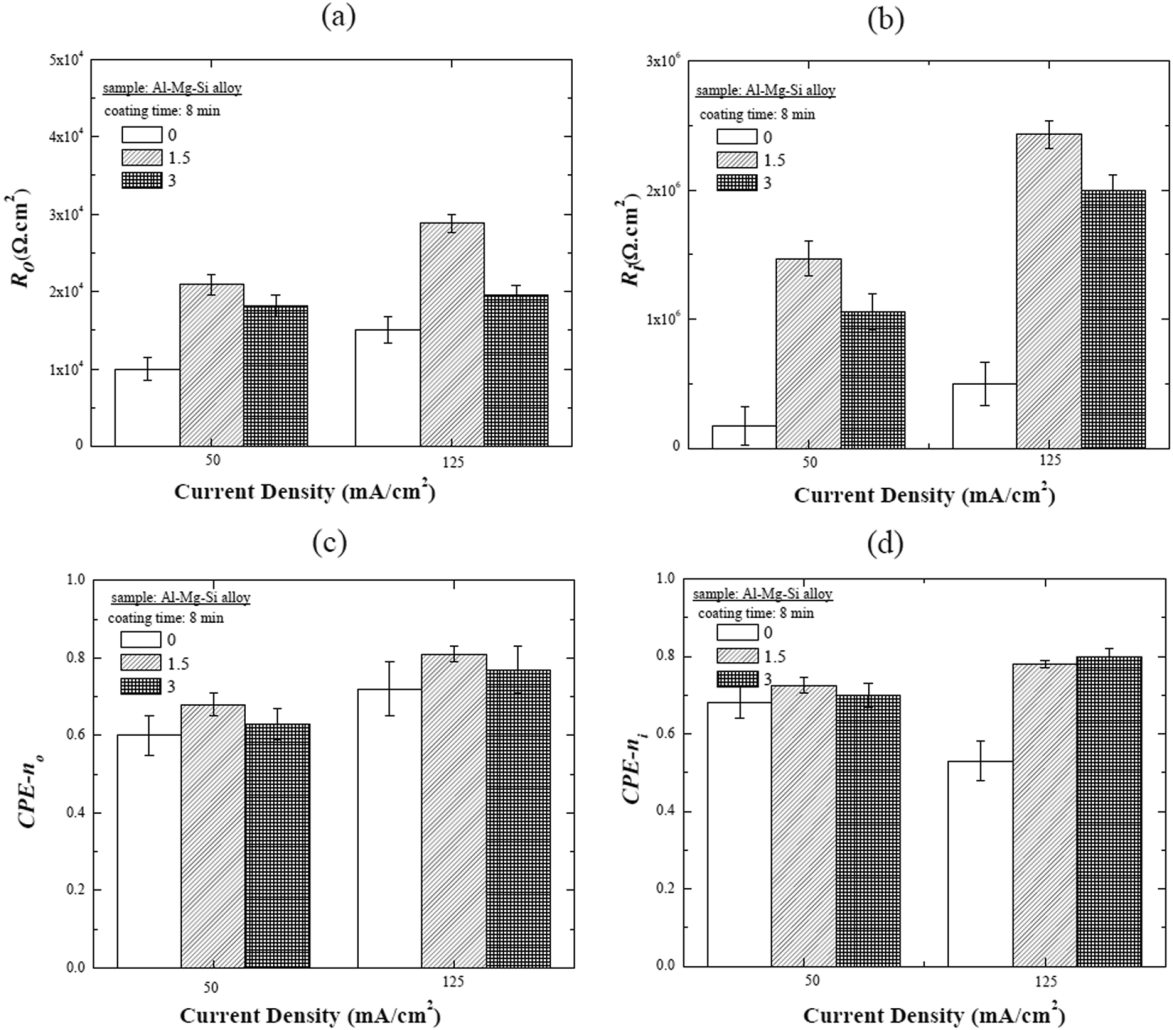
Representation of the EIS-fitted *R*_*0*_, *R*_*i*_, *n*_*0*_, and *n*_*i*_ values of the coatings formed at two different current densities in electrolytes containing different concentrations of Na_3_AlF_6_.

Based on the EIS results and the structure of the PEO coatings, the data was adequately fitted to the equivalent circuit model shown in Fig. 6c ^29^. To better describe the interfacial heterogeneities of the coatings, the more general constant phase element (*CPE*) was used instead of a rigid capacitive element^30^. The *CPE* is defined by the following equation:

5 $${Z}_{CPE}=1/[Y{(j\omega )}^{n}]$$

where *j* is the imaginary unit, *ω* is the angular frequency, and *n* and *Y* are the *CPE* parameters. The *n* values range from 0 to 1: for *n* = 0, the *CPE* describes an ideal resistor, and for *n* = 1, the *CPE* describes an ideal capacitor. The fitted *R*_*o*_, *R*_*i*_, *n*_*o*_, and *n*_*i*_ values for the coatings obtained at different current densities using various concentrations of Na_3_AlF_6_ are presented in Fig. 7 (R is the resistance, and subscripts “o” and “i” denote the outer and inner layers of the coating, respectively).

In terms of *R*_*o*_, which was inversely proportional to coating porosity, it was observed in Fig. 7a that the coating obtained with 1.5 g/L Na_3_AlF_6_ at 125 mA/cm^2^ exhibited the highest *R*_*o*_ value, while the coating obtained with no Na_3_AlF_6_ at 50 mA/cm^2^ had the lowest, further confirming that in the formation of coatings by PEO, the addition of 1.5 g/L Na_3_AlF_6_ to the electrolyte, as well as applying a higher current density, can improve the anticorrosion properties by significantly decreasing the level of porosity. These results were consistent with the SEM results shown in Fig. 1 and Table 1.

In addition, the results shown in Fig. 7b indicated that *R*_*i*_ was generally higher than *R*_*o*_, indicating that the inner layer contributed the most to the overall corrosion resistance. It was also observed that the *R*_*i*_ values for the coatings obtained in electrolytes containing Na_3_AlF_6_ were higher than for those obtained with no Na_3_AlF_6_, suggesting that Na_3_AlF_6_ also produced an increase in the compactness of the PEO coatings through the incorporation of F-containing compounds.

Furthermore, *CPE-n*_*o*_ and *CPE-n*_*i*_ were used as an indicator to confirm microstructural results, such as porosity and micropore size, which strongly affect the anticorrosion properties of PEO coatings^31, 32^. As shown in Fig. 7c and d, the *CPE-n*_*o*_ and *CPE-n*_*i*_ values for the coatings obtained in electrolyte containing 1.5 g/L Na_3_AlF_6_ at 125 mA/cm^2^ were higher than for the other coatings, which indicated that the coating/electrolyte interface and coating/substrate interface became smoother with the addition of 1.5 g/L Na_3_AlF_6_ and upon increasing the current density for the PEO process.

### Mechanism underlying a role of Na_3_AlF_6_

The effect of adding Na_3_AlF_6_ to the alkaline phosphate-based electrolyte on the coating morphology and corrosion resistance was found to be significant. During the PEO process, the AlF_6_^3−^ ions generated due to ionization of Na_3_AlF_6_ were hydrolyzed, which resulted in the formation of Al(OH)_3_ and F^−^ ions (Equation 2). The high temperature under plasma conditions caused dehydration of Al(OH)_3_, which resulted in the formation of Al_2_O_3_ within the coating (Equations 3 and 4)^33^. Moreover, irrespective of current density, the coatings formed in electrolyte containing 3 g/L Na_3_AlF_6_ exhibited a higher F content than their counterparts obtained with 1.5 g/L Na_3_AlF_6_, which suggested that a further hydrolysis of AlF_6_^3−^ ions occurred with 3 g/L Na_3_AlF_6_. It is worth mentioning that the presence of numerous structural defects (Figs 1a and d and 2a and d) in the coatings obtained in the electrolyte with no Na_3_AlF_6_ could provide a short path for F^−^ ions to cross the PEO coating and distribute almost uniformly throughout it under plasma conditions (Fig. 3g). As described earlier, F^−^ ions, being the ion with the smallest size among all negative ions, could enter the micropores, changing the structure and properties of the PEO coatings^27^. Therefore, in comparison, the coatings obtained in electrolyte containing Na_3_AlF_6_ exhibited a relatively defect-free structure. However, compared with the coating obtained with 1.5 g/L Na_3_AlF_6_, the coating obtained with 3 g/L Na_3_AlF_6_, despite having the highest F content, showed larger micropores and a higher degree of porosity, which was attributed to the strong corrosive effect of the electrolyte due to formation of HF^34^. Indeed, a high concentration of HF could lead to the formation of numerous defects in the coating due to a decrease of the electrolyte pH, which produced etching of the substrate surface. In addition, for the coating formed in electrolyte containing 3 g/L Na_3_AlF_6_, some cracks were also observed in the higher magnification (inset of Fig. 1f), which was associated with the transformation of Al(OH)_3_ into Al_2_O_3_. In summary, it was concluded that Na_3_AlF_6_ could lead to an increase in the compactness of the inner layer up to a certain level, above which compactness decreased.

## Conclusions

A nearly defect-free coating formed by plasma electrolytic oxidation at high current density of 125 mA/cm^2^ was obtained in a phosphate-based electrolyte with Na_3_AlF_6_, and the role of Na_3_AlF_6_ in improving the corrosion performance of the coating was investigated. The microstructural results revealed that the coating formed in the electrolyte containing 1.5 g/L Na_3_AlF_6_ at 125 mA/cm^2^ exhibited the structural defects to the less extent in comparison to the other coatings since fluorine was distributed uniformly throughout the coating due to the hydrolysis of AlF_6_^3−^ triggered by intense plasma sparks. Hence, the coating formed in the electrolyte containing Na_3_AlF_6_ would improve the corrosion performance of Al-based alloys.

## Materials and Methods

The substrate sample used in this study was an Al-Mg-Si alloy with a chemical composition (expressed in wt.%) of 0.99 Mg, 0.59 Si, 0.25 Cu, 0.16 Fe, 0.11 Cr, 0.13 Mn, and balance Al. Before the PEO treatment, the initial samples, with a dimension of 25 mm × 20 mm × 4 mm, were ground to 1200 grit with SiC paper and then, ultrasonically cleaned in acetone. The electrolytes were prepared by mixing 3 g/L potassium hydroxide (KOH) and 6 g/L sodium phosphate (Na_3_PO_4_) in distilled water, followed by the addition of Na_3_AlF_6_ at two different concentrations: 1.5 g/L and 3 g/L. The Na_3_AlF_6_ concentration was set at a maximum value of 3 g/L because higher concentrations could cause severe electrochemical etching of the substrate surface. For reference, an electrolyte containing no Na_3_AlF_6_ was also prepared. To obtain the coating, the PEO treatment was conducted at a frequency of 60 Hz during 8 min. In order to study the effect of current density on the characteristics of the coating, two different current densities, viz. 50 and 125 mA/cm^2^, were applied. During the PEO process, the temperature of the electrolyte was controlled to be below 25 °C by adjusting the flow rate of the cooling water. The surface morphology and elemental composition of the coating layers was studied with a scanning electron microscope (SEM, Hitachi S-4800) equipped with an energy dispersive spectrometer (EDS). The Image Analyzer 1.33 program was used to measure and calculate the micropore size and the percentage of porosity. The phase composition was examined by X-ray diffraction (XRD, Rigaku D/Max-2500) with a step size of 0.05° over a scan range from 20° to 90°. The corrosion performance of the PEO coatings was evaluated through potentiodynamic polarization and EIS (electrochemical impedance spectroscopy) tests in a 3.5 wt.% NaCl solution using a Reference 600 potentiostat from Gamry Instruments. A standard three-electrode cell with a coated alloy sample as the working electrode, Ag/AgCl (sat. KCl) as the reference electrode, and a platinum plate as the counter electrode was used in the experiments. Polarization curves were recorded from −0.25 V to +0.4 V with respect to the open circuit potential at a scan rate of 1 mV/s. Impedance measurements were performed on the PEO coatings in the frequency range from 10^6^ Hz to 0.1 Hz with an amplitude of ±10 mV. In order to stabilize the open circuit potential, all electrochemical tests were performed after a 5-h immersion in the 3.5 wt.% NaCl solution. In addition, all tests were repeated at least three times to ensure data accuracy.

## References

[1] Yao Z (2016). Investigation of absorptance and emissivity of thermal control coatings on Mg–Li alloys and OES analysis during PEO process. Sci. Rep..

[2] Mori Y, Koshi A, Liao J (2016). Corrosion resistance of plasma electrolytic oxidation layer of a non-ignitable Mg-Al-Mn-Ca magnesium alloy. Corros. Sci..

[3] Kamil MP, Kassem M, Ko YG (2017). Soft plasma electrolysis with complex ions for optimizing electrochemical performance. Sci. Rep..

[4] Dong K, Song Y, Shan D, Han EH (2015). Corrosion behavior of a self-sealing pore micro-arc oxidation film on AM60 magnesium alloy. Corros. Sci..

[5] Zhao J, Xie X, Zhang C (2016). Effect of the graphene oxide additive on the corrosion resistance of theplasma electrolytic oxidation coating of the AZ31 magnesium alloy. Corros. Sci..

[6] Lu X, Blawert C, Zheludkevich ML, Kainer KU (2015). Insights into plasma electrolytic oxidation treatment with particle addition. Corros. Sci..

[7] Yerokhin AL, Nie X, Leyland A, Matthews A, Dowey SJ (1998). Plasma electrolysis for surface engineering: Review. Surf. Coat. Technol.

[8] Sankara Narayanan TSN, Park S, Lee M (2014). Strategies to improve the corrosion resistance of microarc oxidation (MAO) coated magnesium alloys for degradable implants: prospects and challenges. Prog. Mater. Sci..

[9] Malayoglu U, Tekin KC, Shrestha S (2010). Influence of post-treatment on the corrosion resistance of PEO coated AM50B and AM60B Mg alloys. Surf. Coat. Technol..

[10] Kassem M, Lee YH, Ko YG (2016). Incorporation of MoO_2_ and ZrO_2_ particles into the oxide film formed on 7075 Al alloy via micro-arc oxidation. Mater. Lett..

[11] Rapheal G, Kumar S, Scharnagl N, Blawert C (2016). Effect of current density on the microstructure and corrosion properties of plasma electrolytic oxidation (PEO) coatings on AM50 Mg alloy produced in an electrolyte containing clay additives. Surf. Coat.Technol..

[12] Lu. X, Blawert C, Scharnagl N, Kainer KU (2013). Influence of incorporating Si_3_N_4_ particles into the oxide layer produced by plasma electrolytic oxidation on AM50Mg alloy on coating morphology and corrosion properties. J. Magnes. Alloys..

[13] Kazanski B, Kossenko A, Zinigrad M, Lugovkoy A (2013). Fluoride ions as modifiers of the oxide layer produced by plasma electrolytic oxidation on AZ91D magnesium alloy. Appl. Surf. Sci..

[14] Wang L, Chen L, Yan Z, Wang H, Peng J (2009). Effect of potassium fluoride on structure and corrosion resistance of plasma electrolytic oxidation films formed on AZ31 magnesium alloy. J. Alloys Compd..

[15] Ryu HS, Hong SH (2009). Effects of KF, NaOH, and KOH Electrolytes on Properties of Microarc-Oxidized Coatings on AZ91D Magnesium Alloy. J. Electrochem. Soc..

[16] Liang J (2005). Effect of potassium fluoride in electrolytic solution on the structure and properties of microarc oxidation coatings on magnesium alloy. Appl. Surf. Sci..

[17] Rehman ZR, Koo BH (2016). Combined effect of long processing time and Na_2_SiF_6_ on the properties of PEO Coatings formed on AZ91D. J. Mater. Eng. Perform..

[18] Duan H, Yan C, Wang F (2007). Effect of electrolyte additives on performance of plasma electrolytic oxidation films formed on magnesium alloy AZ91D. Electrochim. Acta..

[19] Yerokhin AL, Lyubimov VV, Ashitkov RV (1998). Phase formation in ceramic coatings during plasma electrolytic oxidation of aluminum alloys. Ceram. Int..

[20] Srinivasan PB, Liang J, Blawert C, Stormer M, Dietzel W (2009). Effect of current density on the microstructure and corrosion behavior of plasma electrolytic oxidation treated AM50 magnesium alloy. Appl. Surf. Sci..

[21] Yue Y, Hua W (2010). Effect of current density on corrosion resistance of micro-arc oxide coatings on magnesium alloy. Trans. Nonferrous Met. Soc. China..

[22] Gao Y, Yerokhin AL, Matthews A (2013). DC plasma electrolytic oxidation of biodegradable cp-Mg: *In-vitro* corrosion studies. Surf. Coat. Technol..

[23] Zhang XL, Jiang ZH, Yao ZP, Wu ZD (2010). Electrochemical study of growth behavior of plasma electyrolytic oxidation coating on Ti6Al4V: Effects of the additive. Corros. Sci..

[24] Kaseem M, Kamil MP, Kwon JH, Ko YG (2015). Effect of sodium benzoate on corrosion behavior of 6061 Al alloy processed by plasma electrolytic oxidation. Surf. Coat.Technol..

[25] Dehnavi V, Liu XY, Luan BL, Shoesmith DW, Rohani S (2014). Phase transformation in plasma electrolytic oxidation coatings on 6061 aluminum alloy. Surf. Coat. Technol.

[26] Malayoglu U, Tekin KC, Malayoglu U, Shrestha S (2011). An investigation into the mechanical and tribological properties of plasma electrolytic oxidation and hard-anodized coatings on 6082 aluminum alloy. Mater. Sci. Eng. A..

[27] Wang Z, Wu L, Cai W, Shan A, Jiang Z (2010). Effects of fluoride on the structure and properties of microarc oxidation coating on aluminum alloy. J. Alloys. Compd..

[28] Arunnellaiappan T, Ashfaq M, Krishna LR, Rameshbabu N (2016). Fabrication of corrosion-resistant Al2O3-CeO2 composite coating on AA7075 via plasma electrolytic oxidation coupled with electrolphoretic deposition. Ceram. Int..

[29] Liu Y, Wei Z, Yang F, Zhang Z (2011). Environmental friendly anodizing of AZ91D magnesium alloy in alkaline borate–benzoate electrolyte. J. Alloys Compd..

[30] Yao Z, Jiang Z, Zin S, Sun X, Wu X (2005). Electrochemical impedance spectroscopy of ceramic coatings on Ti-6Al-4V by micro-plasma oxidation. Electrochim. Acta..

[31] Kaseem M, Ko YG (2016). Electrochemical response of Al_2_O_3_-MoO_2_-TiO_2_ oxide films formed on 6061 Al alloy by plasma electrolytic oxidation. J. Electrochem. Soc..

[32] He D (2014). Effect mechanism of ultrasound on growth of micro-arc oxidation coatings on A96061 aluminum alloy. Vacuum..

[33] Al Bosta, M. M., Ma, K. & Chien, H. Effect of anodic current density on characteristics and low temperature IR emissivity of ceramic coating on aluminum 6061 alloy prepared by microarc oxidation. *J. Ceram*. **2013** (2013).

[34] Gnedenkov SV (2000). Production of hard and heat-resistant coatings on aluminum using a plasma micro-discharge. Surf. Coat.Technol..

